# Timepoint of aspiration can impact diagnostic of PJI - synovial fluid analyses exhibit a high intraindividual variation in periprosthetic joint infections of the knee

**DOI:** 10.1007/s00402-025-05888-8

**Published:** 2025-04-29

**Authors:** Stephanie Kirschbaum, Clemens Gwinner, Tobias Freitag, Michael Schnetzer, Carsten Perka, Michael Fuchs

**Affiliations:** 1 Center for Musculoskeletal Surgery, Charité - University Medicine Berlin, Berlin, Germany; 2 University Department of Orthopaedics RKU, University of Ulm, Ulm, Germany

**Keywords:** Biomarker, Joint aspiration, Synovial leucocyte count, Polymorphonuclear percentage, Septic revision TKA, Periprosthetic joint infection

## Abstract

**Background:**

Synovial leukocyte cell count (LC) and polymorphonuclear percentage (PMN%) are key parameters in the diagnostic workup of periprosthetic joint infection (PJI). Despite ongoing debates regarding optimal thresholds—particularly for reliably excluding low-grade infections—no study has investigated potential confounders such as intra-individual, time-dependent variability of LC and PMN% in patients with PJI following total knee arthroplasty (TKA).

**Methods:**

We conducted a retrospective, double-centre study using prospectively collected data from 35 consecutive patients with confirmed knee PJI, each of whom underwent two joint aspirations at different time points (t1 and t2; mean interval 33 ± 31 days, range 0–111 days; total 70 samples). We analysed absolute and percentage changes in LC and PMN% between t1 and t2 and identified the number of “false-negative” results potentially caused by this variability. A subset analysis was performed on cases exhibiting more than 20% variability to minimize methodological bias. Additionally, we compared LC and PMN% levels between infections caused by low- vs. high-virulence pathogens.

**Results:**

Mean LC among all samples was 19.969 n/µl ± 27.035 n/µl [300–135.000 n/µl], mean PMN% was 65%±28% [6-97%]. Seven (20%) of the LC results and four (11%) of the PMN% results exhibited discrepant values, either falling below or exceeding the threshold depending on the timepoint of aspiration. Regarding the time-dependent variability, there was a trend to higher percentual change for LC compared to PMN% (58% ± vs. 11%; *p* = 0.062). Synovial analyses of low-virulent bacteria showed significantly decreased overall LC- and PMN%-values compared to high virulent pathogen associated PJI (*p* < 0.001).

**Conclusion:**

LC values exhibit significant intra-individual variability of up to 58% over time, which could lead to false-negative exclusion of PJI in up to 20% of cases if only a single aspiration were performed. In contrast, PMN% is less affected by timing, showing a variability of just 11%. Notably, in PJI caused by low-virulence pathogens, both LC and PMN% show approximately 80% variability, whereas PMN% variability in high-virulence infections is only 22%. LC, in particular, demonstrates a high degree of independent variability. These findings suggest that synovial LC—especially—is less reliable diagnostically than previously assumed. PMN%, on the other hand, appears to be less influenced by timing and pathogen virulence, and may therefore be the more robust parameter. In addition to timing, future research should explore other potential confounders that may impact the diagnostic reliability of both LC and PMN%.

**Level of evidence:**

III.

**Supplementary Information:**

The online version contains supplementary material available at 10.1007/s00402-025-05888-8.

## Introduction

The diagnostic workup of periprosthetic joint infections (PJI) comprises various parameters such as clinical examinations, laboratory markers, microbial testing as well as histopathological examinations [20]. Synovial analysis of joint aspirates, including leucocyte cell count (LC) and polymorphonuclear percentage (PMN%) have become the diagnostical gold-standard of PJI evaluation [6]. Regarding different PJI classification systems, certain cutoff-values for LC and PMN% remain the subject of an ongoing scientific debate being redefined several times within the last years [7, 16, 23, 28, 33]. To distinct aseptic from septic total knee arthroplasty (TKA) failures, earlier publications defined a cut-off value for LC of 1,700–2,500 n/µL and a polymorphonuclear percentage of 60–65% [31, 34]. More recent classification systems consider a LC of > 3,000 cells/µL and a PMN% of > 80% as confirmed PJI [21, 23]. Despite numerous studies investigating the ideal cut-off for PJI diagnosis, interestingly, none of the existing literature explores intra-individual variability as a potential challenge or confounder. For example, a recent report indicates that intra-individual LC and PMN% in aseptic TKA failures exhibit considerable time-dependent variation [10]. Unfortunately, there is no data investigating intraindividual variance of LC and PMN% in patients with PJI of the knee. Therefore, the present study aims to evaluate the level of intraindividual variation in LC and PMN and examine whether PMN% shows less variance compared to LC in septic cases.

## Materials and methods

In this double-center study, we conducted a retrospective analysis of prospectively collected data to determine the variation of LC and PMN% in patients with confirmed PJI of the knee. The study protocol was approved by the local ethics committee (registration number: 40/20– FSt/Sta).

### Patients’ enrollment

**Fig. 1:**
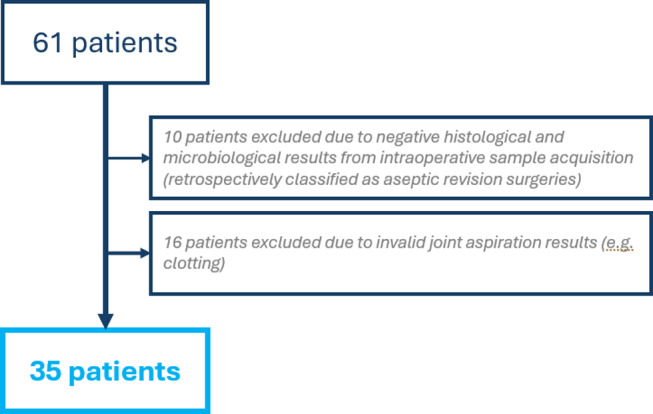
Flowchart pf patients’ enrolment

All patients receiving septic revision surgery of their TKA between December 2018 to July 2020 were reviewed retrospectively. PJI was defined according to the Musculoskeletal Infection Society (MSIS) definition criteria [23].The inclusion criterion was a minimum age of 18 years, septic revision surgery of TKA (Debridement, Irrigation and Implant Retention (DAIR), septic 1-stage or 2-stage TKA revision) and two valid joint aspirations with a maximum time period of 120 days in-between. Exclusion criteria included revision surgery due to aseptic TKA failure or antibiotic treatment for any reason prior to or between the two joint aspirations. Furthermore, patients with rheumatic or autoimmune diseases, those receiving immunosuppressive therapy, or those who underwent an additional externally performed joint aspiration or intra-articular injection between the evaluated time points were excluded. These factors might influence synovial leukocyte count (LC) and polymorphonuclear percentage (PMN%), thus representing relevant confounders in the interpretation of these parameters [29, 32]. In addition, patients with early postoperative PJI (within 12 weeks after primary implantation) were excluded, as LC levels are physiologically elevated during this period, making it difficult to differentiate between infection-related elevation and postoperative inflammatory responses, such as hematoma-associated increases [26]. Patients with incomplete documentation, invalid results of joint aspiration, missing intraoperative microbiological or histological samples were further excluded from the recent study (*n* = 16, Fig. 1). Furthermore, 10 patients with elevated LC and PMN%, which were highly suggestive for PJI according to MSIS criteria, showed negative microbiological and histopathological findings. Due to substantial polyethylene wear or failure of metallosis, which are known causes for elevated synovial LC beside PJI, these cases were classified as aseptic and therefore excluded from the study [1, 3].

In total, 35 patients (20 women, 15 men) underwent confirmed septic revision of their TKA and offered complete data including two valid joint aspirations. The median age at the time of surgery was 71 ± 13 years (range, 39–94 years). Six patients (17.1%) received a DAIR procedure, 11 patients (31.4%) a one-stage and 18 individuals (51.4%) a two-stage septic revision surgery.

### Timepoint and technique of joint aspiration

Each patient underwent joint aspiration of the knee at two different timepoints. The first sample collection of synovial fluid was performed in the outpatient department. The second joint aspiration was performed in the operating room after skin incision and subcutaneous preparation via intraarticular joint puncture at the beginning of surgery. The average time between the first and second joint aspiration was 33 ± 31 days [0–111 days]. There were no saline-assisted aspirations, or any local anesthetic agents injected before sample collection. Moreover, to prevent potential confounding effects on LC and PMN%, no antibiotics were given prior to any joint aspiration. Synovial fluid aspirations were preserved and transported to the laboratory within 6 h.

### Cell count analyzation

All cell counts were generated automatically using a special analyzer (ABX Micros 60, Horiba Medical, Montpellier, France). Given that automated cell counts in body fluids generally demonstrate an imprecision of less than 10%, but can reach up to 20% in low-cellularity fluids [2], a substantial change was defined as a deviation in LC of greater than 20% between time points t1 and t2. A sub-analysis focusing on this cohort was performed in addition to the overall analysis.

### Microbiological findings and PJI category

Furthermore, intraoperatively collected microbiological as well as histological samples were used to categorize infections as low-, or high virulent pathogen associated PJI or culture-negative PJI according to the study of Krenn et al. and Boyle et al. [5, 19]. Differences in average LC and PMN% as well as LC- and PMN%- variability between these categories were evaluated. In cases of polymicrobial infections or different grades of virulence between pre- and intraoperative cultures, the higher virulence was chosen for classification in the recent study.

### Statistical analysis

Statistical Analysis was performed using Statistical Package für Social Sciences (SPSS - Version 26, SPSS Inc., Chicago, IL, USA). Data was expressed as mean ± standard deviation and or median (interquartile range) according to its distribution. The results of the percentage-based evaluation were rounded for improved readability. Shapiro Wilk Test was used to test Gaussian distribution. The comparison of the non-parametric data was tested by a Wilcoxon Signed Rank Test or Man-Whitney U-test. Pearson’s chi-square test was used to determine statistically significant differences between categorical variables. *P* < 0.05 was considered statistically significant. A sub-analysis of (1) cases demonstrating a variability of more than 20% LC between aspirations and (2) between low-and high-virulent pathogen associated PJI was conducted. Furthermore, a post-hoc analysis of LC as well as PMN% variability was conducted using G-Power version 3.1.9.6 (Franz Faul, University of Kiel). For the power analysis individual intraindividual synovial LC as well as PMN% change between first and second aspiration was used instead of mean LC and PMN% of all first and second aspirations in order to avoid any bias due to summarizing but to contribute to the intraindividual time-dependent changes. Since the data were not normally distributed, the Wilcoxon test was applied. Given that LC and PMN% could increase or decrease in the second aspiration, a two-tailed approach was used. Post-hoc analysis showed a power of 78% using the mean intraindividual difference in synovial LC (11,630, STAW 23,800, resulting in effect size of 0.488, sample size 35 and alpha error 0.05) and a power of 99% concerning mean intraindividual PMN% changes (10.5%, STAW 10.5%, resulting in effect size of 1, sample size 35 and alpha error 0.05).

## Results

### Overall data

**Fig. 2:**
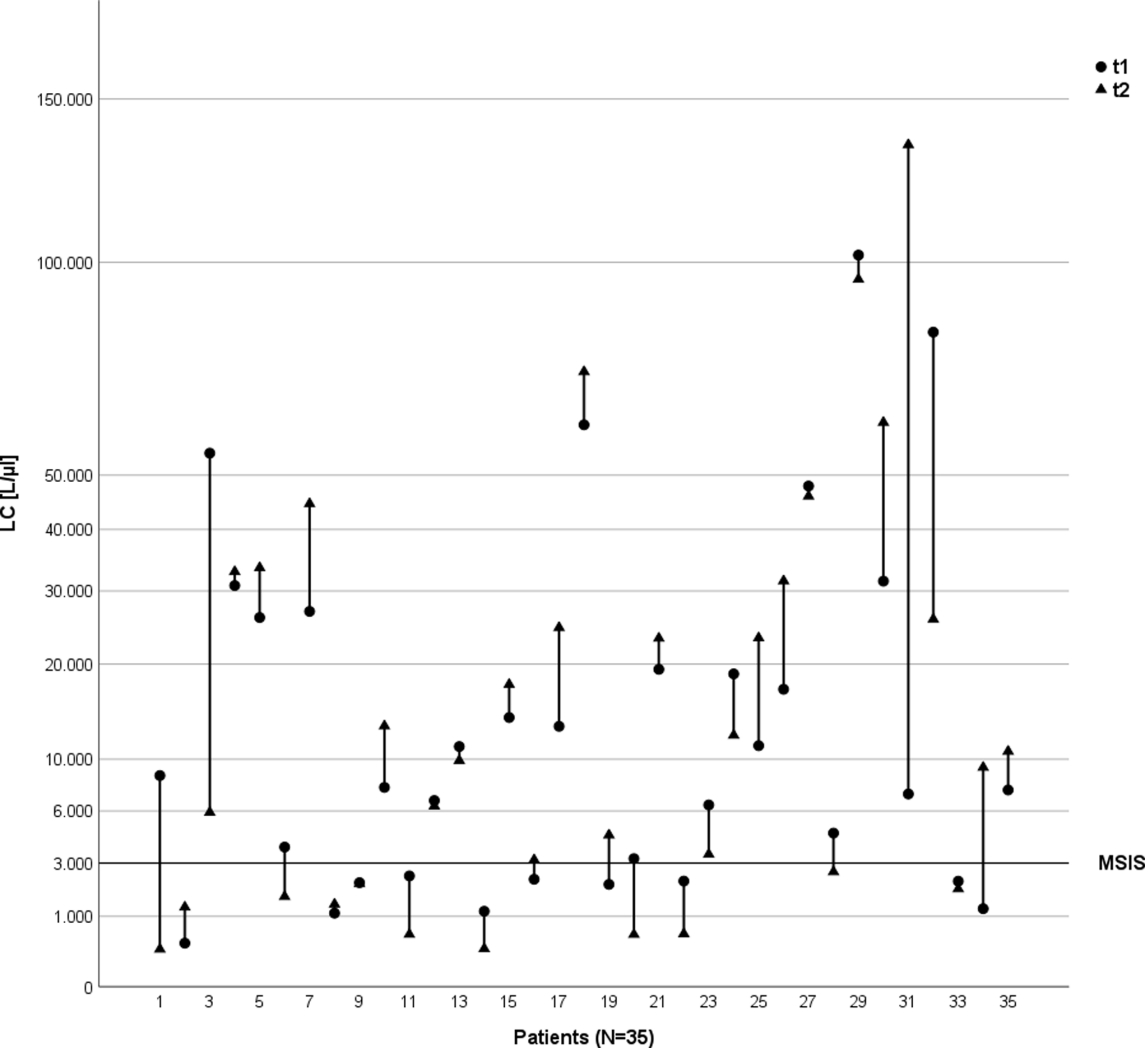
Variance of Leucocyte Cell Counts (LC). Respective LC values (n/µL) per patient at the first (t1) and second (t2) joint aspiration. The displayed line illustrates the cut-off level suggestive for PJI according to the MSIS criteria (LC ≥ 3,000/µL)

**Fig. 3:**
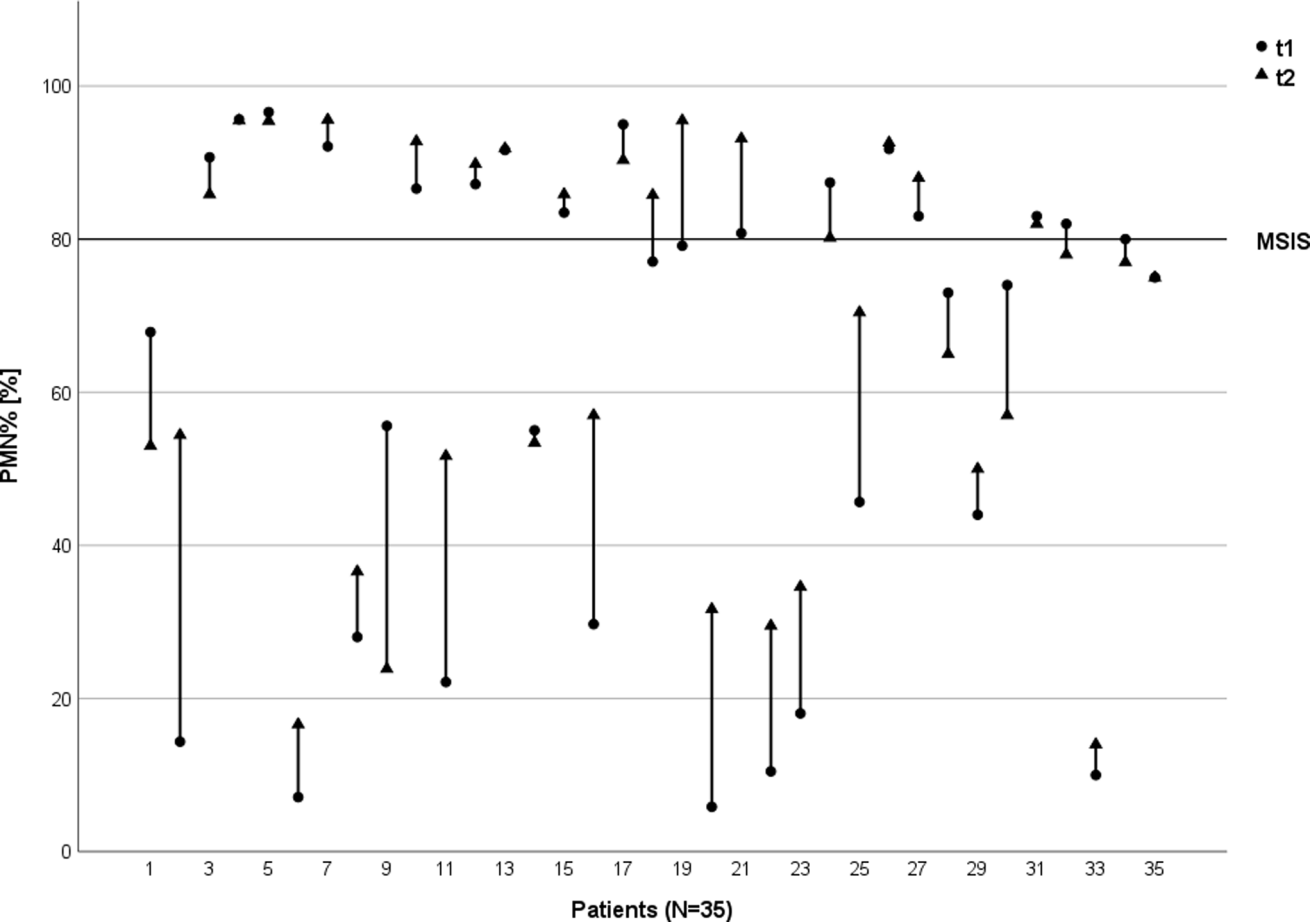
Variance of Polymorphonuclear Percentage (PMN%). Respective PMN% values per patient at the first (t1) and second (t2) joint aspiration. The displayed line illustrates the cut-off level suggestive for PJI according to the MSIS criteria (PMN% ≥ 80%)

In total, seventy synovial fluid samples were analyzed. Mean LC of all 70 samples was 19,969 n/µl ± 27,035 n/µl [300–135,000 n/µl], mean PMN% was 65% ± 28% [6 − 97%]. LC levels unsuggestive for infection were observed in 20 of 70 joint aspirations (29%), irrespective of the joint aspiration date (Fig. 2). For PMN%, 38 out of 70 joint aspirations (54%) were below the PJI-associated threshold of 80% (Fig. 3).

### Change of septic revision character depending on aspiration time point

**Fig. 4:**
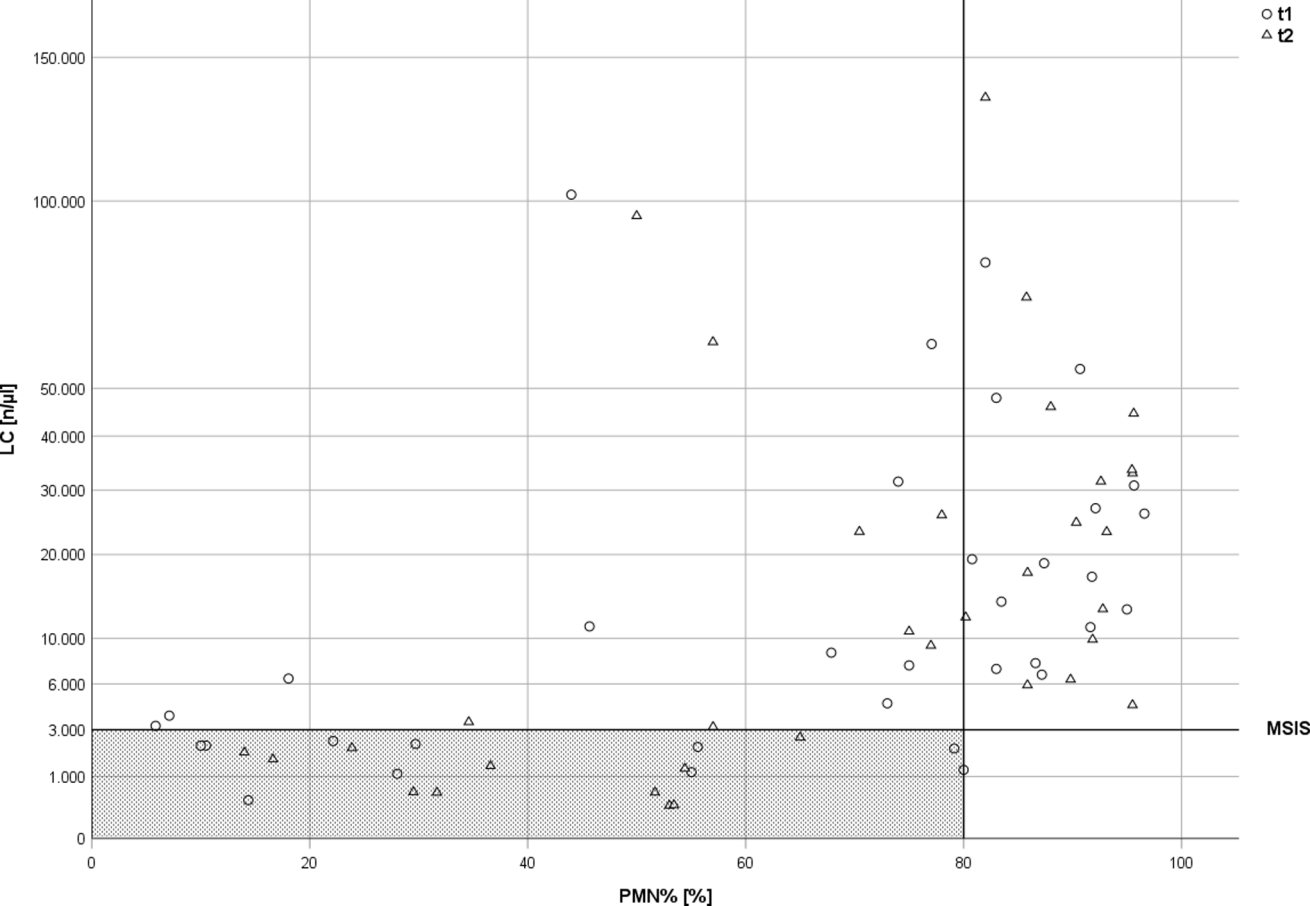
Different Leucocyte Cell Counts (LC) and Polymorphonuclear Percentage (PMN%) of all patients with displayed MSIS criteria. LC (n/µL) and PMN% at the respective timepoints of the first (t1) and second (t2) joint aspiration. Displayed lines illustrate the cut-off levels suggestive for PJI according to the PJI classification systems. The grey-shaded area represents all results below the defined thresholds

In seven cases (20%) LC results showed discrepant values indicating PJI as being either below or above the threshold of 3,000 leucocytes /µl depending on the aspiration timepoint. For PMN%, this was evident in 4 patients (11%) (Figs. 2, 3 and 4).

### Intraindividual variance of LC and PMN

There were no significant differences between first and second LC and PMN% values, despite the median percentual variability was 58% [2 − 1,775] for LC and 11% [0-442] for PMN% (Table 1, *p* = 0.062). There was no relation between the aspiration time interval of t1 and t2 and percentual change of LC (Pearson = -0.186, *p* = 0.221) but a moderate correlation between timespan and percentual change of PMN% (Pearson = 0.471, *p* = 0.004).

**Table 1:** Overall comparison of Leucocyte Cell Count and Polymorphonuclear Percentage between first and second aspiration

	1.Aspiration	2.Aspiration	Mean intraindividual difference	Significance
LC [n/µl]	18,262 ± 24,061 7,695 [397 − 102,000]	21,676 ± 29,972 9,887 [300 − 135,000]	11,630 ± 23,800 3,200 [45–127,800]	0.272
PMN [%]	63 ± 31 77 [6–97]	68 ± 26 77 [14–96]	11 ± 11 6 [0–40]	0.053

Data presented as average ± SD and Median [range] as not distributed normally. Leucocyte Cell Count (LC), Polymorphonuclear Percentage (PMN%)

### Sub-analysis of cases with > 20% variability of LC

Twenty-seven of 35 cases (77.1%) demonstrated a relevant change of LC > 20%. There was no significant difference between first and second LC and PMN% values, despite the median percentual variability was 73% [20 − 1,775] for LC and 11% [0-442] for PMN% (Table 2).

**Table 2:** Sub-analysis and comparison of Leucocyte Cell Count and Polymorphonuclear Percentage between first and second aspiration in cases with percentual Leucocyte Cell Count change > 20% between aspirations

	1.Aspiration	2.Aspiration	Mean intraindivi-dual difference	Significance
LC [n/µl]	13,924 ± 18,251 7500 [397 − 81,700]	18,217 ± 27,988 9300 [300 − 135,000]	14,168 ± 26,587 3950 [273 − 127,800]	0.195
PMN [%]	61 ± 32% 75 [6–97]	68 ± 24 75 [17–96]	12 ± 11 8 [0–40]	0.069

Data presented as average ± SD and Median [range] as not distributed normally. Leucocyte Cell Count (LC), Polymorphonuclear Percentage (PMN%)

Again, there was no relation between time interval and percentual change of LC (Pearson = -0.273, *p* = 0.168) but a moderate correlation between timespan and percentual change of PMN% (Pearson = 0.456, *p* = 0.017).

### Comparison of low- vs. high- virulent pathogen associated PJI

Seventeen cases (48.6%) were identified as low-virulent pathogen caused PJI, 18 cases (51.4%) as high-virulent pathogen caused PJI. The average LC was significantly higher in high-virulent associated PJI than in low-virulent pathogen associated PJI (33,382 n/µl ± 31,913 [397 − 135,000] vs. 5767 n/µl ± 6630 [300 − 23,340], *p* < 0.001). In line with PMN% (82% ±18 [14–97] vs. 48% ± 27 [6–93], *p* < 0.001).

Variability of PMN% was significant less in high-virulent pathogen associated PJI (*p* = 0.008,Table 3). Supplemental material provides an overview about all cases depending on pathogen virulence.

**Table 3:** Comparison of Leucocyte Cell Count and Polymorphonuclear Percentage variability (in absolute and percentual change) between first and second aspiration depending on low- and high-virulent pathogen associated periprosthetic joint infection

	low-virulent	high-virulent	Significance
Leucocyte Cell Count (LC)			
Variability in %	88 ± 151 46 [2-662]	155 ± 408 67 [4 − 1,775]	0.987
Polymorphonuclear Percentage (PMN%)			
Variability in %	77 ± 108 40 [6-441]	22 ± 65 5 [0-279]	0.008

Data presented as average ± SD and Median [range] as not distributed normally. Leucocyte Cell Count (LC), Polymorphonuclear Percentage (PMN%)

## Discussion

The diagnosis of periprosthetic joint infection remains to be challenging. Despite ongoing debates regarding ideal cut-off values which are currently ranging between 1,600-3,200/µl and 66–76% of PMN [7, 14, 17, 30, 33], synovial LC and PMN% are considered first-line diagnostic tests [16, 23, 31]. In light of the ongoing discussion about optimal diagnostic thresholds, it is noteworthy that no study to date has evaluated potential confounding factors such as the timing of aspiration, the effect of NSAIDs as anti-inflammatory agents as well as immunomodulatory medication on synovial LC counts, or the impact of physical activity prior to aspiration. This study is the first investigating a possible cofounding factor and demonstrate significant intra-individual, time-dependent variability—particularly in LC counts—in patients with confirmed PJI. Specifically, both low- and high-virulence pathogen-associated PJI exhibited similar intra-individual and time-dependent variations in LC and PMN%. According to the classification, this variability could alter the diagnostic outcome from “confirmed PJI” to “no PJI” or “PJI unlikely” in 11% of cases when considering combined LC and PMN% values.

Several factors could explain these biomarker variations. First, the homeostasis of PJI-affected joints is subject to continuous inflammatory signaling, resulting in varying cytokine-mediated immunological responses potentially leading to different biomarker concentrations. In addition, the intake of non-steroidal anti-inflammatory drugs or also immunomodulatory medication could also influence this cytokine-mediated immune response and thus also have an indirect influence on the cell count in the synovial fluid. Only one older study evaluates the effect of corticosteroid or NSAIDs uptake on synovial fluid after 24 h. Despite the significant reduction of inflammatory markers (especially prostaglandin E2) no change was seen concerning synovial LC [27]. Authors concluded that LC count is not influenced within 24 h but assumed potential effect in case of longer medication as inflammatory markers (prostaglandin E2) correlate with synovial LC. As NSAID intake was no primary outcome parameter in the recent study patient information concerning amount and timepoint of intake was typically incomplete. Therefore, unfortunately no correlation between LC and PMN% and NSAID medication was possible within this study. However, no studies have been published to date regarding the impact of NSAID intake in the diagnostic of PJI so this might be an interesting topic for future research. Immune Modifying drugs also seem to reduce the synovial cell count by reducing inflammatory markers and gene expression [29, 32]. Therefore, it seems logical, that high-virulence pathogens cause higher LC and PMN% values than low-virulent ones as demonstrated in result section. In line, Renz et al. found that the sensitivity of several biomarkers in Cutibacterium acnes-related implant infections, a classic low-virulence pathogen, is very poor [24]. Interestingly, the degree of LC and PMN% variation in low-grade PJI was comparable to that in patients with aseptic total knee arthroplasty failure (LC 44.1%, SD 28.6% vs. PMN% 27.3%, SD 23.7%) [10]. This suggests also a physical impact on biomarker levels, as leukocytes are affected by gravitation and sedimentation. Thus, joint motion and activity before aspiration might impact laboratory results. To date, no studies, except the mentioned one on aseptic cases, have evaluated biomarker variance relative to time or activity level before joint aspiration.

If each synovial analysis were evaluated independently, our study revealed that 29% of all aspirations did not exceed established LC and PMN% cut-off levels, despite confirmed PJI by microbiological and histopathological results. This underscores the ongoing debate regarding biomarker thresholds. Gramlich et al. evaluated 405 patients with suspected PJI of TKA, hip (THA) or shoulder arthroplasty and found that the optimal diagnostic cut-offs for LC and PMN% were 2,479/µL and 67%, respectively [12]. Zahar et al. suggested even lower cut-offs, with 1,630 leukocytes/µL for TKA and 3,063 leukocytes/µL for THA [33]. Gallo et al. recommended 4,100 leukocytes/µL for THA and 3,200 leukocytes/µL for TKA [11]. Despite varying LC thresholds, recommended PMN% were similar for hip and knee in the above mentioned studies: Zahar et al. recommended PMN% of 60.5% for TKA and 66.1% for THA, Gallo 72.8% for TKA and 76.5% for THA [11, 33]. These findings further suggest that PMN% might offer greater diagnostic accuracy than LC. Our study supports this hypothesis: considering both aspirations, 20% of PJIs would have been missed by LC alone, compared to 11% by PMN% alone. Heckmann et al. found that intraarticular saline lavage significantly lowered synovial white blood cell count, but PMN% remained stable, further supporting this hypothesis [13].

Given the ongoing debate regarding optimal thresholds and potential confounders such as time-dependent variability for synovial LC and PMN% in diagnosing periprosthetic joint infection (PJI), emerging diagnostic methods appear particularly important in borderline cases. Alpha-defensin (sensitivity 96%, specificity 95% at 5.2 mg/L, D-lactate (sensitivity 90.7%, specificity 83.3% at 0.04 mmol/L), and S-Pecam (sensitivity 82%, specificity 80% at 54.3 ng/mL have shown promise in enhancing diagnostic accuracy [4, 8, 9, 18]. Additionally, next-generation sequencing (NGS) may improve pathogen detection in up to 50% of culture-negative PJI cases, underscoring the value of these complementary tools [15].

This study has several limitations. It is a retrospective analysis of prospectively collected data, subject to inherent design limitations. The sample size was relatively small, and 16 out of 61 patients with suspected PJI were excluded due to invalid or missing laboratory analyses. However, post-hoc power analysis revealed sufficient power. Even more noteworthy is that 10 out of original 61 patients, initially diagnosed as septic due to elevated cell counts, exhibited aseptic results in histological and microbiological tests. This highlights, on one hand, the limitations of relying solely on synovial fluid analysis for diagnosis and, on the other hand, underscores the need for enhancing preoperative diagnostic criteria in PJI like described above. Even though there were no significant differences with respect to LC- and PMN% variation in relation to the time interval between both aspirations, the interval was heterogeneous, influenced by patient health and the urgency of septic revision TKA. Additionally, natural variation in quantitative laboratory analyses introduces some degree of technical variability [22, 25]: equipment-based inaccuracies could contribute, with deviations up to 20% reported, particularly in low-cellularity synovial fluid [2]. However, a sub-analysis in the present study ruled out systematic error, confirming PMN% as a reliable marker with consistent intraindividual variability (11%). Despite these note-worthy technical challenges, which might lead to slight differences, our findings underscore the importance of PMN% in diagnosing PJI.

## Conclusion

Both gold-standard diagnostic markers for PJI (synovial LC and PMN%) exhibit significant intra-individual variability influenced by time and organism virulence, which undoubtedly affects their assumed diagnostic accuracy. By comparison, LC should be regarded as less reliable than PMN% as it expresses higher intra-individual changes over time. Orthopaedic surgeons should be aware of the possibility of time-depending and therefore false-negative results (risk 11–20%). Therefore, a comprehensive diagnostic approach—including clinical assessment, blood tests, microbiology, synovial analysis, and histology—should be employed. Additionally, emerging diagnostic tools, such as pathogen-specific biomarkers and reverse transcription-quantitative polymerase chain reaction (RT-qPCR), should be considered in ambiguous cases [21, 23]. Furthermore, studies investigating potential confounders, such as the impact of physical activity on sedimentation effects or NSAID use and its potential suppression of the immune-inflammatory response—and consequently LC and PMN%—are needed. Such research is essential not only to establish quantitative thresholds for PJI diagnosis but also to define qualitative parameters.

## Electronic supplementary material

Below is the link to the electronic supplementary material.

Supplementary Material 1

## Data Availability

Data is provided within the manuscript or supplementary information files.

## Abbreviations

PJI: Periprosthetic joint infection
LC: Leucocyte cell count
PMN%: Polymorphonuclear percentage
TKA: Total knee arthroplasty
THA: Total hip arthroplasty
MSIS: Musculoskeletal Infection Society
DAIR: Debridement, Irrigation and Implant Retention
